# MYBL1 induces transcriptional activation of ANGPT2 to promote tumor angiogenesis and confer sorafenib resistance in human hepatocellular carcinoma

**DOI:** 10.1038/s41419-022-05180-2

**Published:** 2022-08-20

**Authors:** Jinrong Zhu, Yongqi Wu, Yijian Yu, Yan Li, Jianfei Shen, Rongxin Zhang

**Affiliations:** 1 Guangdong Province Key Laboratory for Biotechnology Drug Candidates, School of Life Sciences and Biopharmaceutics, Guangdong Pharmaceutical University, Guangzhou, China; 2 Department of Cardiothoracic Surgery, Taizhou Hospital of Zhejiang Province affiliated to Wenzhou Medical University, Taizhou, China

**Keywords:** Tumour angiogenesis, Transcriptional regulatory elements

## Abstract

Angiogenesis is considered as an important process in tumor growth, metastasis of hepatocellular carcinoma (HCC) and associated with cancer progression, suggesting that an important research and development field of clinical molecular targeted drugs for HCC. However, the molecular mechanisms underlying tumor angiogenesis in HCC remains elusive. In the current study, we demonstrate that upregulation of AMYB proto-oncogene-like 1 (MYBL1) was associated with high endothelial vessel (EV) density and contributed to poor prognosis of HCC patient. Functionally, MYBL1 overexpressing enhanced the capacity of HCC cells to induce tube formation, migration of HUVECs, neovascularization in CAMs, finally, enhanced HCC cells metastasis, while silencing MYBL1 had the converse effect. Furthermore, HCC cells with high MYBL1 expression were more resistance to sorafenib treatment. We observed that CD31 staining was significantly increased in tumors formed by MYBL1-overexpressing cells but decreased in MYBL1-silenced tumors. Mechanistically, MYBL1 binds to the ANGPT2 promoter and transcriptionally upregulate ANGPT2 mRNA expression. Strikingly, treatment with monoclonal antibody against ANGPT2 significantly inhibited the growth of MYBL1-overexpressing tumors and efficiently impaired angiogenesis. Furthermore, the histone post-translational factors: protein arginine methyltransferase 5 (PRMT5), MEP50, and WDR5 were required for MYBL1-mediated ANGPT2 upregulation. Importantly, we confirmed the correlation between MYBL1 and ANGPT2 expression in a large cohort of clinical HCC samples and several published datasets in pancreatic cancer, esophageal carcinoma, stomach adenocarcinoma, and colon cancer. Our results demonstrate that MYBL1 upregulated the ANGPT2 expression, then induced angiogenesis and confer sorafenib resistance to HCC cells, and MYBL1 may represent a novel prognostic biomarker and therapeutic target for patients with HCC.

## Introduction

Hepatocellular carcinoma (HCC) is the most frequent primary liver cancer and is the third leading cause of cancer-related deaths worldwide, with 10% for the 5-year overall survival rates for locally advanced patients and 3% for metastatic disease [1–3]. HCC appears frequently in patients with diverse chronic liver diseases underlying cirrhosis and is one of the highly vascularized solid tumors characterized by arterialization of its blood supply and vascular abnormalities [4, 5]. Although the current treatment of HCC remains an arduous task due to lacking the effective systemic therapy, the status of angiogenesis in HCC correlates with the disease progression and prognosis sheds light antiangiogenic therapy on a promising novel treatment for this disease [6, 7]. Hence, exploration of the molecular mechanisms and identification of a novel therapeutic target for angiogenesis to improve HCC prognosis is urgent need.

The protein arginine methyltransferase 5(PRMT5) is responsible for regulation of multiple biological processes including transcription, RNA splicing, metabolic signaling, differentiation, and spliceosome assembly [8, 9]. PRMT5 always complexed with the WD-repeat protein MEP50 and binding to the catalytic site to produce methylarginine of histone or target protein [10, 11]. H3R2me1 or H3R2me2s modified by PRMT5-mediated transcriptional activation is always recruited the WDR5 methyltransferase complexes, subsequently induced histone H3K4me3 which is recognized by the RNA polymerase II transcription complex on the promoters of target genes [12]. Abnormal expression of PRMT5, MEP50, or WDR5 are found in many cancers and often correlated with malignant progression of tumor indicates that PRMT5/MEP50/WDR5 complexes plays an important role in cancer progression.

Sorafenib repressed tumor growth by inhibiting vascular endothelial cell membrane receptor VEGFR1/2 and reducing angiogenesis in tumor tissue [13]. Although VEGFR is the main growth-promoting signal pathway in vascular endothelial cells, the driving factors of angiogenesis also include angiopoietin (ANG)-tie, placental growth factor (PlGF), and transforming growth factor β [14]. Tumor vascular endothelial cells can maintain angiogenesis by activating growth factors independent of VEGF/VEGFR signaling pathway, which leads to the clinical drug resistance of sorafenib. Angiopoietin-2 (ANGPT2) is a secreted factor which belonging to the angiopoietin/Tie (tyrosine kinase with Ig and EGF homology domains) signaling pathway, and has been reported to play a central role in diseases related to blood-vessel growth under certain physiological and pathological circumstances [15–17]. Numerous studies have demonstrated that ANGPT2 mostly explored in tumor-induced angiogenesis, where its overexpression or inhibition increased or decreased the angiogenesis capability, respectively, suggesting a critical role for ANGPT2 in angiogenesis [18–21]. Consistently, the expression of ANGPT2 has been reported to be correlated with microvessel density and tumor size and metastatic efficacy, which suggested ANGPT2 an independent prognostic factor in the progression of multiple cancer types [22–25]. Importantly, encouraging results have recently shown that ANGPT2 inhibition restored vascular stability, repressed tumor angiogenesis, and decreased the metastatic burden and proportion of sorafenib tolerance of early or even advanced-stage malignancies [26–28]. Therefore, illumination the molecular mechanisms of deregulation of ANGPT2 may provide new approaches for the development of targeted cancer therapies.

V-myb myeloblastosis viral oncogene homolog (MYB) proteins belong to a large family of transcription factors and functionally diverse, such as cancer initiation and progression, immunological disease or developmental disorders, and represented in all eukaryotes [29, 30]. MYB proto-oncogene-like 1 (MYBL1), preferred named myb-related protein A, belongs to the family of the myb proto-oncogene family and was cloned from a B lymphocyte cDNA library of the missing 3′ half of the human A-myb cDNA [31, 32]. It has been reported that MYBL1 functions as a strong activator of transcription containing a conserved DNA binding homeodomain and transcriptional activation domain [31]. Numerous studies have demonstrated that MYBL1 is highly expressed in many cancers and involved in malignant development of tumor. Audrey Player et al. discovered and validated MYBL1 as one of the six candidate genes in TNBC cell lines with respect to the expression of the cytokine IL32 [33]. Brayer and colleagues reported that MYB and MYBL1 recurrent fusions define a common transcription factor-driven oncogenic pathway in salivary gland adenoid cystic carcinoma by RNA sequencing analysis [34]. Although Xie et al. previously demonstrated that MYBL1 promotes growth and metastasis of HCC cells via transcriptionally upregulated TWIST1 expression [35]. However, the clinical significance and molecular mechanisms of MYBL1 underlying tumor angiogenesis and sorafenib resistance in cancer remain elusive.

In this study, we reported that MYBL1 enhances the expression of ANGPT2 via directly targeting the ANGPT2 promoter and transcriptionally upregulate ANGPT2 mRNA expression via interacting with PRMT5, MEP50, and WDR5, subsequently promotes HCC angiogenesis and enhanced sorafenib resistance both in vitro and in vivo. Therefore, our results uncover a novel mechanism for upregulation of ANGPT2 in cancer and indicate an oncogenic role for MYBL1 in HCC angiogenesis and sorafenib resistance.

## Materials and methods

### Cell lines and cell culture

HCC cell lines (HepG2 and Hep3B) were purchased from ATCC (Manassas, VA, USA) and grown in Dulbecco’s modified Eagle’s medium (DMEM, Invitrogen) supplemented with 10% FBS (Gibco) at 37 °C with 5% CO_2_. All cell lines were authenticated by short tandem repeat (STR) fingerprinting.

### Patient information and tissue specimens

In our study, we randomly selected 88 paraffin-embedded HCC samples from the tissue bank of Taizhou Hospital of Zhejiang Province. The clinical features were evaluated by professional pathologists and were showed in Supplementary Tables S1–2. The present study was approved by the Institutional Research Ethics Committee of Taizhou Hospital of Zhejiang Province for research purposes.

### Plasmids, virus constructs, and retroviral infection of target cells

Coding sequence of human MYBL1 was cloned into the pCDH-CMV-MCS-3Flag-EF1-CopGFP-T2A-Puro vector by multiple cloning sites. PLKO-U6-EGFP-P2A-PURO vector was used to generate MYBL1-RNAi#1, MYBL1-RNAi#2. Lipofectamine 3000 reagent (Invitrogen, Carlsbad, CA) was used to transfect MYBL1 plasmids or shRNA according to the manufacturer’s instruction. Primers of overexpression and knockdown is indicated in supplementary Tables S3.

### Western blot analysis

Total proteins were harvested from indicated cells and separated by SDS/PAGE gel and then transferred to PVDF membranes for 1 h. Subsequently, membranes were blocked by using 5% non-fat milk powder and then using the primary antibodies, anti-flag (Sigma, F3165), anti-MYBL1 (Affinity, AF9007), anti-ANGPT2 (Abcam, ab155106), anti-HA (Proteintech, 51064-2-AP), anti-myc (Proteintech, 16286-1-AP) and anti-PRMT5 (Proteintech, 18436-1-AP). Immunoreactive proteins were detected using a fluorescence imaging system (Minichemi 610, China).

### Immunohistochemistry (IHC)

The paraffin-embedded tissues are placed in a 60° oven to bake the slices for 40 min to melt the paraffin. The tissues were then treated with 30% H_2_O_2_ for 30 min and Microwave repair with 0.01 M sodium citrate buffer with pH 6.0. Subsequently, paraffin-embedded tissues were blocking with serum and analyzed using IHC with anti-MYBL1, anti-ANGPT2, anti-Ki67, or anti-CD31 antibody and finally hematoxylin stain, then gradient alcohol and xylene dehydration, mount. The scoring method was used for immunohistochemical analysis according to previous studies [36]. In short, the tissue sections were scored according to the degree of staining (0–3 points are negative coloring, light yellow, light brown, and dark brown) and the positive scale (1–4 points are 0–25%, 26–50%, 51–75%, and 76–100%), Finally, the scores was calculated as the product of degree of staining and the positive scale. The above analysis methods were reviewed and performed double-blind analysis by two independent pathologists.

### Statistics

We use SPSS 21.0 statistical software to analyze the data of our study. Statistical tests including log-rank test, Chi-square test, Student’s two-tailed *t* test, Spearman’s rank correlation coefficients, and univariate and multivariate Cox regression analyses. Data represent mean ± SD. *P* values < 0.05 were considered statistically significant.

### Microarray data process and visualization

Microarray data were downloaded from the TCGA database: (http://www.tcga.org/) and from GEO database: (https://www.ebi.ac.uk/arrayexpress/);

For analysis the Venn diagram: http://bioinformatics.psb.ugent.be/webtools/Venn/.

GSEA was performed using GSEA 2.0.9: (http://www.broadinstitute.org/gsea/).

## Results

### Upregulation of MYBL1 combine with high EV density was associated with HCC patient prognosis

**Fig. 1:**
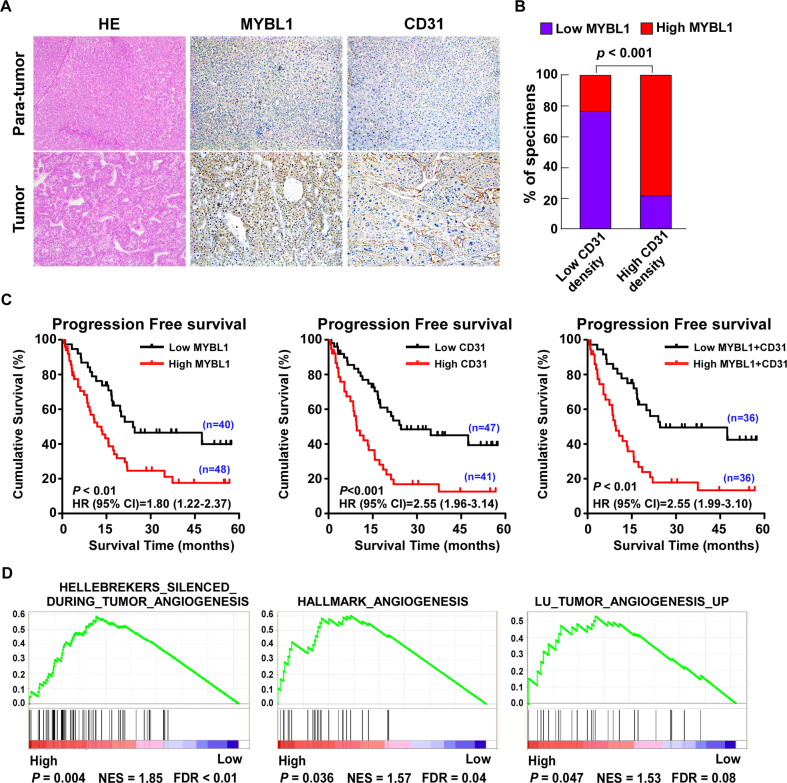
Upregulation of MYBL1 correlates with high EV density and associates with HCC patient prognosis. **A** IHC staining indicating the MYBL1 and CD31 protein expression in para-tumor tissues and HCC tissues. **B** MYBL1 were positively correlated with CD31 protein levels in clinical specimens. **C** Patients with high MYBL1 expression (left), CD31 density (middle), and both MYBL1 and CD31 expression (right) show progression-free survival in HCC patients. **D** The correlation analysis between the mRNA levels of MYBL1 and angiogenesis in TCGA datasets.

To identify the relationship between MYBL1 expression and angiogenesis in HCC, we examined the expression of MYBL1 and the CD31 staining to measure (endothelial vessel) EV density. As shown in Fig. 1A, we firstly found that expression of MYBL1 was higher in HCC tissues, compared with para-tumor tissues. We also found that MYBL1 expression was upregulated in high EV density tissues compared with its level in low EV density tissues (Fig. 1A). Moreover, the levels of MYBL1 were positively correlated with CD31 protein levels (Fig. 1B, *p* < 0.01). Kaplan–Meier analyses revealed that the high level of MYBL1 (*p* < 0.01, HR (95% CI) = 1.80 (1.22–2.37)), high CD31 staining (HR (*p* < 0.001, 95% CI) = 2.55 (1.96–3.14)) and grouped by both MYBL1 expression and CD31 staining (*p* < 0.01, HR (95% CI) = 2.55 (1.99–3.10)) were associated with reduced progression-free survival time in HCC patients (Fig. 1C). Furthermore, MYBL1 overexpression significantly correlated with gene signatures associated with angiogenesis (HELLEBREKERS_SILENCED_DURING_TUMOR_ ANGIOGENESIS, HALLMARK_ANGIOGENESIS, LU_TUMOR_ ANGIOGENESIS_UP) in TCGA dataset of HCC, according to gene set enrichment analysis (GSEA) (Fig. 1D). The above results suggesting that MYBL1 was overexpressed in HCC and MYBL1 expression combined with EV density was associated with HCC patient prognosis.

### Ectopic expression of MYBL1 promotes HCC angiogenesis in vitro

**Fig. 2:**
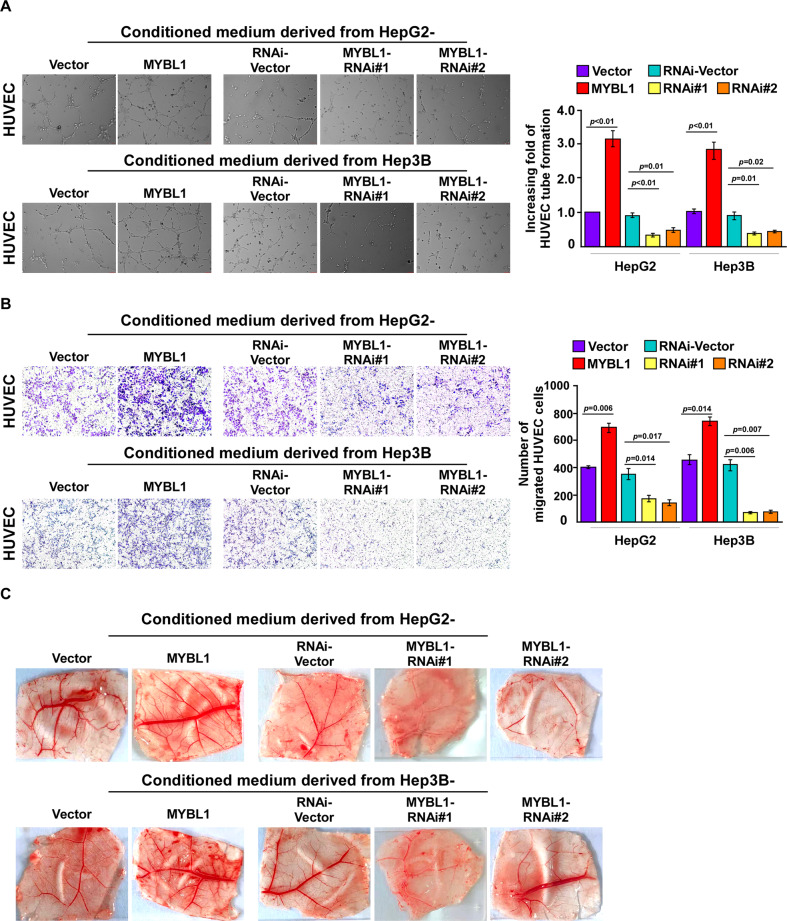
Ectopic expression of MYBL1 promoted HCC angiogenesis in vitro. **A** The effect of MYBL1 on HUVEC tube formation with conditioned media from the indicated HCC cells. **B** The effect of MYBL1 on HUVEC cell migration with conditioned media derived from the indicated HCC cells. **C** Representative images of the blood vessels formed in the CAM assay, after stimulation with conditioned medium from the indicated cells. Each bar represents the mean ± SD of three independent experiments.

Based on the correlation analysis between MYBL1 and EV density, we next studied the biological function of MYBL1 in regulating tumor angiogenesis. HepG2 and Hep3B cells stably expressing MYBL1 cDNA and MYBL1 RNAi(s) were established (Supplementary Fig. S1A). As shown in Fig. 2A, overexpressing of MYBL1 strongly provoked the ability of HCC cells to induce tube formation of Human umbilical vein endothelial cells (HUVECs), but silencing MYBL1 abrogated this phenotype. Migration assays revealed that ectopic expression of MYBL1 significantly induced, whereas silencing MYBL1 reduced the ability of migration of HUVECs (Fig. 2B). Strikingly, the data of chick embryo chorioallantoic membrane (CAM) experiments indicated that overexpression of MYBL1 strongly enhanced, whereas downregulation of MYBL1 inhibited, the abilities of neovascularization (Fig. 2C), suggesting that MYBL1 promotes HCC angiogenesis in vitro tube formation assays revealed that.

### MYBL1 promotes HCC angiogenesis in vivo

**Fig. 3:**
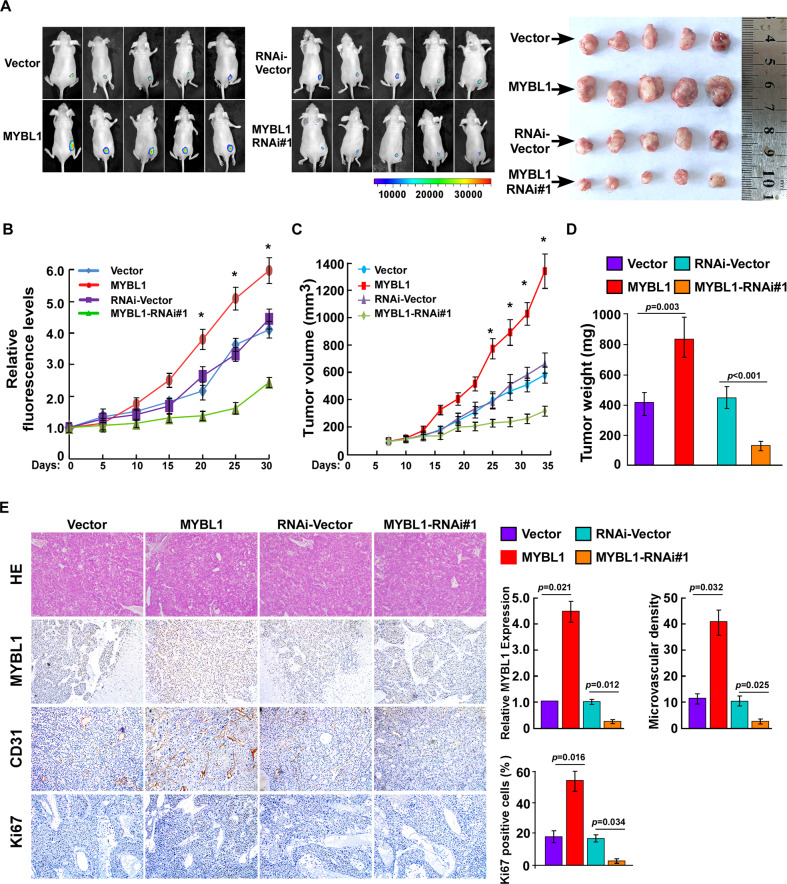
MYBL1 promotes HCC angiogenesis in vivo. **A** HCC cells were subcutaneous injected into nude mice (*n* = 5/group). Fluorescence signal of nude mice was measured (right). Representative images of tumor-bearing mice; Images of the tumors from all mice in each group (left). **B** Quantification of fluorescence signal was measured on the indicated days. **C** Tumor volumes of nude mice from the indicated group were measured. **D** Tumor weights of nude mice from the indicated group were measured. **E** H&E, Ki67and CD31 staining was performed in the indicated group. Each bar represents the mean ± SD of three independent experiments.

Furthermore, the biological role of MYBL1 in HCC angiogenesis was further examined via using an in vivo tumor model. As shown in Fig. 3A, B, the fluorescence intensity formed by HepG2/MYBL1 cells were stronger than the respective control tumors. Furthermore, the tumor formed by HepG2/MYBL1 cells were larger, in both size and weight than the control tumors (Fig. 3C, D). However, the tumors formed by HepG2/MYBL1-RNAi cells were smaller and lighter than the tumors formed by RNAi-vector cells (Fig. 3C, D). Importantly, we found that the Ki67 positive ratio and the EV density, as indicated by the number of CD31-positive vessels, was significantly increased in HepG2/MYBL1 tumors, and decreased in HepG2/MYBL1-RNAi tumors (Fig. 3E), suggesting that MYBL1 contributes to HCC angiogenesis in vivo. Furthermore, the transwell matrix penetration assay showed that MYBL1 enhanced the capacity of migrated and invasive of HCC cells than vector control cells, whereas MYBL1-inhibited cells had a lower migrated and invasive capacity (Supplementary Fig. S1B, C). Collectively, the above dates strongly suggest a vital role of MYBL1 as an angiogenesis inducer and promoted motility and invasiveness in HCC cells.

### MYBL1 upregulates *ANGPT2* in HCC cells

**Fig. 4:**
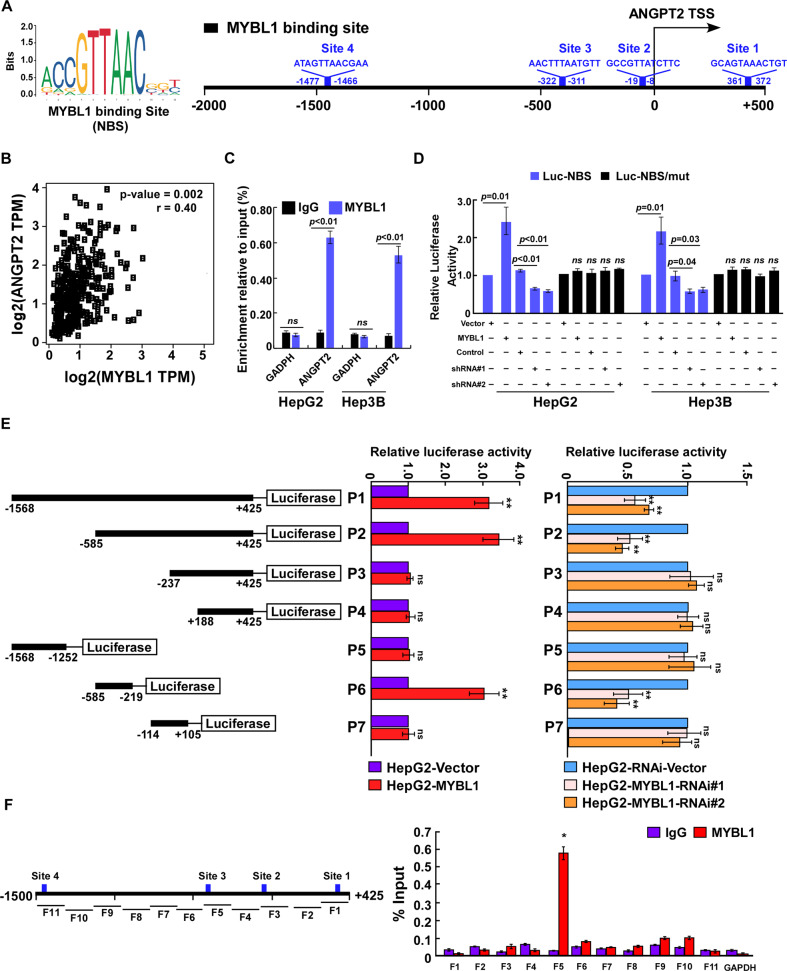
MYBL1 upregulates ANGPT2 in HCC cells. **A** Four potential MYBL1 binding sites were predicted in the promoter of ANGPT2 using the JASPAR database. TSS: Transcription start site. **B** Correlation analysis of MYBL1 and ANGPT2 in HCC cells. **C** ChIP analysis of the association between MYBL1 and ANGPT2 gene transcripts in HCC cells. **D** Relative promoter luciferase activities of ANGPT2 in the indicated HCC cells. **E** Left panel: schematic illustration of the cloned fragments of the human *ANGPT2* promoter. The promoter region was cloned as six fragments (P1-P7). Right panel: transactivation activity of the indicated serial *ANGPT2* promoter fragments in the indicated cells. **F** Analysis of the physical association of regions of the *ANGPT2* promoter with MYBL1 using a CHIP assay. Left panel, schematic illustration of PCR-amplified fragments of *ANGPT2* promoter. Right panel, CHIP assays were performed in HCC cells using an MYBL1 antibody to identify *ANGPT2* promoter regions bound to MYBL1. IgG was used as a negative control. Error bars represent the mean ± SD of three independent experiments. *P < 0.05.

It has been well-known that ANGPT2 expression is biologically linked to angiogenesis and metastasis of tumor cells and play an important role in cancer progression. Therefore, we investigate whether ANGPT2 is involved in MYBL1-induced angiogenesis and metastasis in HCC. Interestingly, there are four potential MYBL1-binding sites were predicted in the promoter of ANGPT2 by using the JASPAR database (https://jaspar.genereg.net/) (Fig. 4A). Moreover, the expression of ANGPT2, at both mRNA and protein levels, was consistently upregulated in MYBL1-overexpressing HCC cells, but downregulated in MYBL1-silenced HCC cells compared to control cells (Supplementary Fig. S2A, B). The association of MYBL1 with ANGPT2 was further confirmed by analysis correlation of MYBL1 and ANGPT2 expression in TCGA dataset (*r* = 0.4, *p* = 0.002, Fig. 4B). A ChIP assay demonstrated that MYBL1 was significantly associated with the *ANGPT2* promoter (Fig. 4C). Luciferase reporter assay was performed and showed that overexpression of MYBL1 promoted, while silencing MYBL1 repressed, the luciferase activity of the ANGPT2 promoter in a dose-dependent manner in HCC cells but shown no effect on the luciferase activities of the promoter with a deleted MYBL1-binding site (Fig. 4D and Supplementary Fig. S2C). As illustrated in Fig. 4E and Supplementary Fig. 2D, we found that the luciferase activities driven by serial DNA fragments cloned from the *ANGPT2* promoter region (P1: −1568 to +425; P2: −585 to +425 (P2); P6: −585 to −219, were significantly increased in MYBL1 overexpression cells and decreased in MYBL1 knockdown condition. Additionally, chromatin immunoprecipitation (ChIP) assay revealed that MYBL1 bound to region 6: −585 to −219 within the *ANGPT2* promoter region (Fig. 4F), indicating that MYBL1 transcriptionally upregulates *ANGPT2* by targeting the *ANGPT2* promoter in HCC.

### ANGPT2 is required for MYBL1-induced angiogenesis

**Fig. 5:**
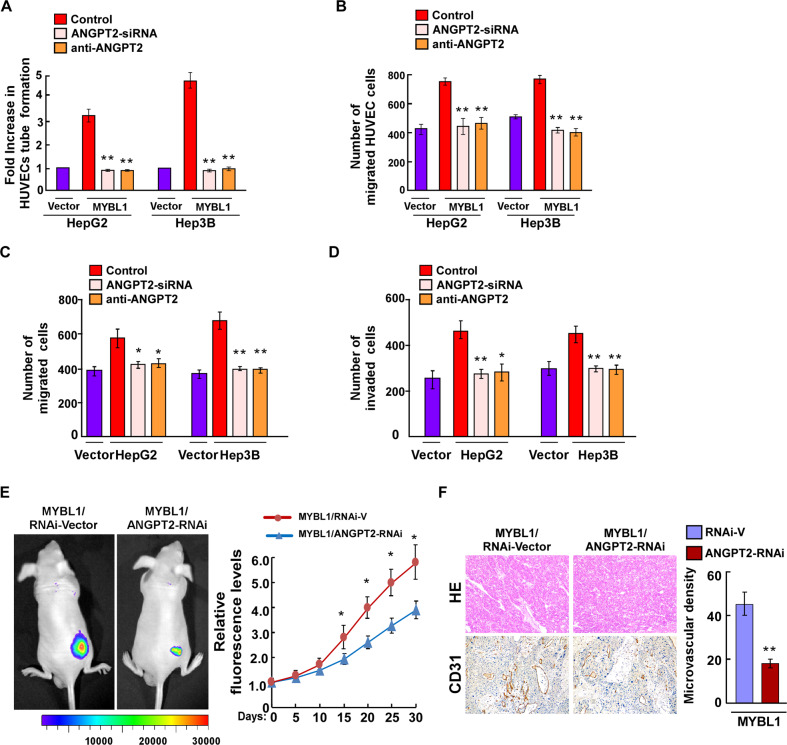
ANGPT2 is required for MYBL1-induced angiogenesis. **A** Quantification of HUVEC tube formation in the indicated group. **B** Cell migration assay was performed by culturing HUVEC with conditioned media derived from MYBL1-transduced HCC cells transfected with *ANGPT2* siRNA or treated with a neutralizing anti-ANGPT2 antibody. **C** Quantification of indicated migration HCC cells by transwell assays. **D** Quantification of indicated invasion HCC cells by transwell assays. **E** Relative fluorescence signal of nude mice was measured on the indicated group. **F** H&E and IHC staining analyses the CD31 expression in the indicated group. Each bar represents the mean ± SD of three independent experiments. *P < 0.05, **P < 0.01.

The in vitro results showed that silencing ANGPT2 significantly decreased the ability of MYBL1-transduced HCC cells to induce HUVEC tube formation and migration (Fig. 5A, B and Supplementary Fig. S3). The anti-ANGPT2 antibody (R&D #623-AN-025, 10 ng/ml) was used to neutralize ANGPT2 and added to the concentrated conditioned medium for 1 h of incubation, subsequently treat HUVECs. Neutralize ANGPT2 significantly decreased the ability of MYBL1-transduced HCC cells to induce HUVEC tube formation and migration (Fig. 5A, B), as well as decreased the ability MYBL1-induced migrated and invasive of HCC cells (Fig. 5C, D). Furthermore, the in vivo results showed that silencing ANGPT2 significantly reduced the fluorescence intensity in MYBL1-overexpression tumor cells (Fig. 5E) and downregulation of ANGPT2 dramatically reduced the number of CD31-positive microvessels in MYBL1 transfected mice (Fig. 5F). Collectively, these results indicate that ANGPT2 is a key mediator of MYBL1-induced HCC angiogenesis.

### The PRMT5/MEP50/WDR5 is involved in MYBL1-induced angiogenesis via epigenetically regulation of ANGPT2

**Fig. 6:**
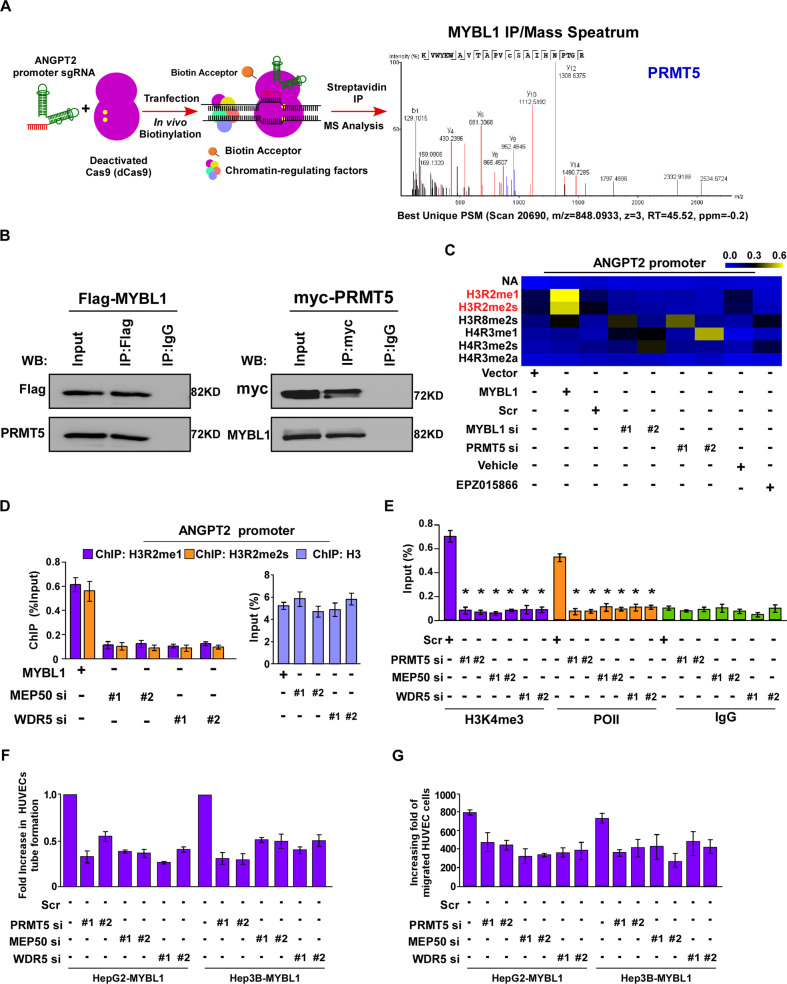
The PRMT5/MEP50/WDR5 is involved in MYBL1-induced angiogenesis via epigenetically regulation of ANGPT2. **A** Schematic of dCas9-mediated capture of chromatin interactions between MYBL1 and PRMT5. **B** Co-IP assay showing that endogenous MYBL1 interacted with endogenous PRMT5 in HCC cells. **C** Heatmap of ChIP-qPCR enrichments of histone post-translational modifications on MYBL1 promoter in the indicated cells. **D** ChIP-qPCR enrichments of H3R2me1 and H3R2me2s on ANGPT2 promoter region in the indicated group. **E** ChIP-qPCR enrichments of H3K4me3, polymerase II, or IgG on ANGPT2 promoter region in the indicated group. **F** Quantification of HUVEC tube formation cultured on Matrigel-coated plates in the indicated group. **G** Cell migration assay was performed by culturing HUVEC with conditioned media in the indicated group.

Furthermore, biotinylated deactivated Cas9 (dCas9) capture analysis combined with Immunoprecipitation and mass spectrometry analysis were performed to explore the mechanism by which MYBL1 transcriptionally activated ANGPT2 in HCC. We found that MYBL1 and PRMT5 were involved in the transcriptional regulation of ANGPT2 in HCC cells (Fig. 6A). Co-immunoprecipitation (co-IP) analyses demonstrated that MYBL1 interacts with PRMT5 in HCC cells (Fig. 6B). It has been reported that PRMT5 plays an important role in transcriptional activation or repression via diverse histone methylation modifications. Herein, we found that MYBL1 significantly increased levels of H3R2me1 and H3R2me2s on the *ANGPT2* promoter, however it was markedly abolished by MYBL1 or PRMT5-silencing (Fig. 6C). Furthermore, we found that inhibiting PRMT5 activity using the PRMT5 inhibitor EPZ015866 significantly reduced the levels of H3R2me1 and H3R2me2s on the *ANGPT2* promoter in MYBL1-overexpression HCC cells (Fig. 6C). The above results indicated that PRMT5-mediated histone methylation contributes to MYBL1-induced transcriptional activation of *ANGPT2*. It has been reported that PRMT5-induced H3R2me1 and H3R2me2s promote transcription by forming a unique hetero-octameric complex, which is composed of four PRMT5 proteins plus four essential cofactors, MEP50 (methylosome protein 50)/WDR6 (WD repeat domain 6). Herein, we demonstrate that silencing PRMT5, MEP50, WDR5 or treatment with small-molecule OICR-9429, an inhibitor of WDR5 interaction with the H3 tail, significantly reduced the levels of H3R2me1 and H3R2me2s on the *ANGPT2* promoter and decrease the mRNA levels of *ANGPT2* in MYBL1-overexpression HCC cells (Fig. 6D and Supplementary Fig. S4A). We also found that inhibition of PRMT5, MEP50, or WDR5 strongly decreased the abundance of H3K4me3 and Polymerase II on the *ANGPT2* promoter in HCC cells (Fig. 6E). Importantly, functionally, silencing PRMT5, MEP50, WDR5 or treatment with OICR-9429 significantly profoundly impaired the ability of MYBL1-induced migrated and invasive of HCC cells and MYBL1-transduced HCC cells to induce HUVEC tube formation and migration (Fig. 6F, G and Supplementary Fig. 4B–E). The above results demonstrated that MYBL1 upregulated the transcription level of ANGPT2 via methylation modification of H3R and subsequent methylation of H3K4 at the ANGPT2 promoter.

### Clinical relevance of MYBL1 and ANGPT2 expression in HCC

**Fig. 7:**
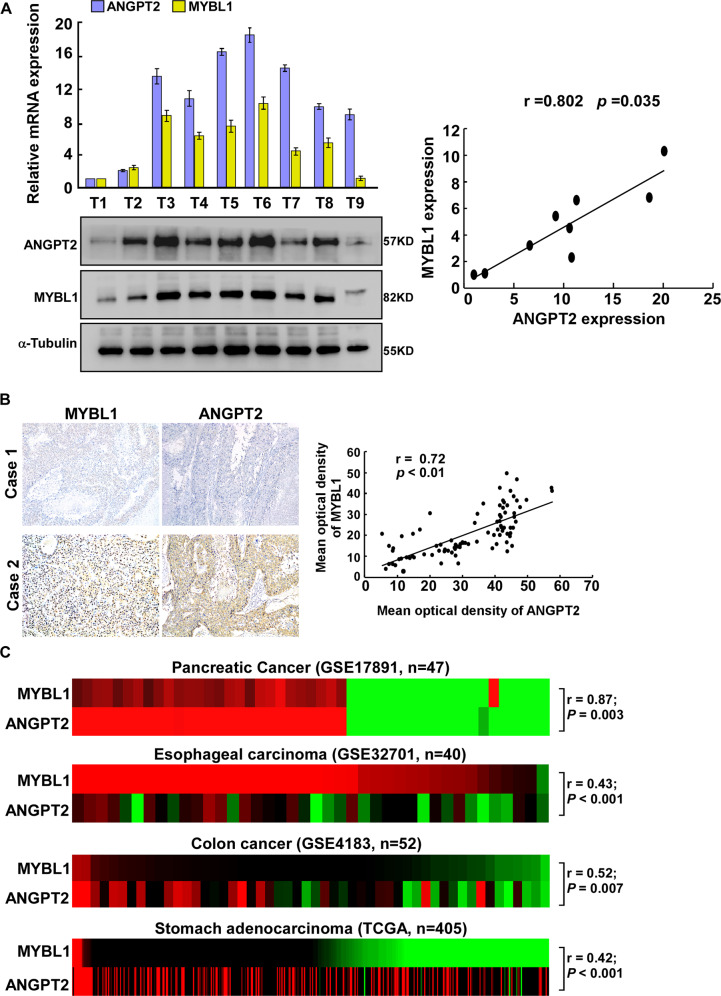
Clinical relevance of MYBL1 and ANGPT2 expression in HCC. **A** Analysis of expression (left) and correlation (right) of MYBL1 with ANGPT2 mRNA expression in 9 freshly collected HCC samples. **B** MYBL1 levels were positively associated with ANGPT2 expression in primary human HCC specimens. Two representative cases are shown. **C** MYBL1 expression correlated positively with ANGPT2 expression in published profiles of multiple cancers. Each bar represents the mean ± SD of three independent experiments.

To examine the clinical relevance of MYBL1 and ANGPT2 expression in HCC, western blot and IHC assay were performed. As shown in Fig. 7A, the expression of MYBL1 in 9 freshly collected clinical HCC samples was positively correlated with the protein expression levels of *ANGPT2* (*r* = 0.802, *P* = 0.035). Concordantly, MYBL1 levels correlated strongly with the expression of ANGPT2 in HCC specimens via performing IHC analysis (Fig. 7B, *p* < 0.01). Furthermore, we found that MYBL1 was overexpression in several gastrointestinal cancers (Supplementary Fig. S5) and the clinical relevance of MYBL1 and ANGPT2 was further confirmed in multiple cancers of published profiles: pancreatic cancer (NCBI/GEO/GSE 17891; *r* = 0.87; *p* = 0.003; *n* = 47); esophageal carcinoma (NCBI/GEO/GSE 32701; *r* = 0.43; *p* < 0.001; *n* = 40); stomach adenocarcinoma (TCGA; r = 0.42; *P* < 0.01; *n* = 405); colon cancer (NCBI/GEO/GSE 4183; *r* = 0.52; *p* = 0.007; *n* = 52) (Fig. 7C).

### MYBL1 overexpression confers sorafenib resistance to HCC cells

**Fig. 8:**
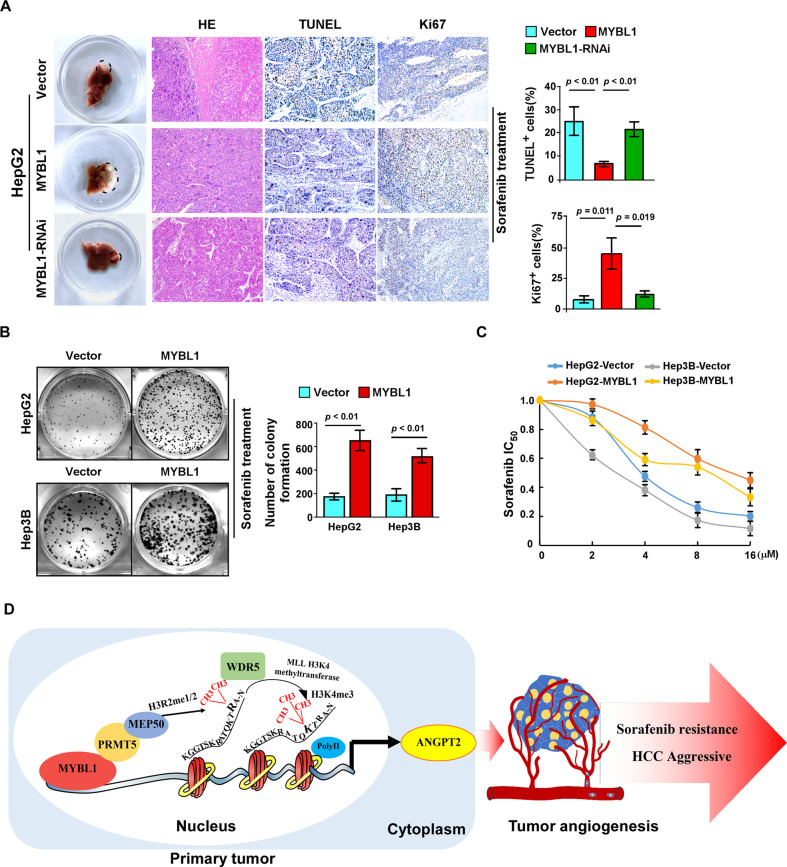
MYBL1 overexpression confers sorafenib resistance to HCC cells. **A** Representative tumor images from liver (left); IHC staining indicating the Tunel and Ki67 protein expression in the indicated group (middle); quantification of Tunel and Ki67 protein expression in the indicated group(right). **B** Representative micrographs (left) and quantification (right) of colony formation of HCC cells. **C** IC50 of sorafenib in the indicated cells. **D** Schematic diagram illustrating that MYBL1 induces transcriptional expression of ANGPT2 to promote tumor angiogenesis and confer sorafenib resistance in human hepatocellular carcinoma.

It has been reported that tumor angiogenesis plays important role in tumor growth and drug resistance. We examined whether MYBL1-induced tumor angiogenesis is involved in the regulation of sorafenib resistance in HCC cells. The in-situ tumor model experiments showed that overexpression of MYBL1 significantly abolished its anti-tumor effect on tumor growth of HCC cells treatment with sorafenib, as evidenced by rapid tumor progression, and lower proportions of TUNEL^+^ and higher proportions of Ki67^+^ cells, however, inhibition of MYBL1 has the opposite effect (Fig. 8A). In agreement with the in vivo results, in vitro assays also demonstrates that overexpression of MYBL1 significantly enhanced sorafenib resistance compared with the vector control in HCC, which was indicated by increased numbers of colony formation cells and an increasing IC50 of sorafenib (Fig. 8B, C) (IC50 values were: HCC-Vector 4.98 μM; HCC-MYBL1 12.60 μM; Hep3B-Vector 2.87 μM; Hep3B-MYBL1 7.79 μM). Taken together, the above results support the notion that MYBL1 overexpression confers sorafenib resistance to HCC cells.

## Discussion

Although recent study has demonstrated that MYBL1 promotes growth and metastasis of HCC cells via upregulated TWIST1 expression [35], however, the clinical significance and molecular mechanisms of MYBL1 underlying tumor angiogenesis in HCC remain remains largely unknown. Herein, our study presents the first demonstration that overexpression of MYBL1 was associated with high endothelial vessel (EV) density in HCC. Upregulation of MYBL1 induces the mRNA expression of ANGPT2 and significantly promotes HCC angiogenesis, enhanced sorafenib resistance both in vitro and in vivo. Mechanistically, MYBL1 directly targeting the ANGPT2 promoter and transcriptionally upregulate ANGPT2 mRNA expression via interacting with PRMT5, MEP50, and WDR5. More importantly, the positive correlation observed between MYBL1 and ANGPT2 expression was not only confirmed in a cohort of human HCC specimens, but also in several published datasets such as pancreatic cancer, gastric cancer, and stomach adenocarcinoma (Fig. 8D). Therefore, our results uncover a novel mechanism for upregulation of ANGPT2 in cancer and indicate an oncogenic role for MYBL1 in HCC angiogenesis and sorafenib resistance.

Clinical and experimental data demonstrate that tumor microvascular density (MVD) is an important prognostic factor of HCC, and angiogenesis contributes to the progression of HCC and is associated with poor prognosis for HCC [37, 38]. In recent years, clinical anti-angiogenesis therapy has been proved to be an effective treatment strategy for many human cancers, especially for HCC, a typical hypervascularized tumor. The standard therapeutic approach, transarterial chemoembolization (TACE), was now widely used in the treatment of HCC by emblazing the blood supply artery of liver cancer, resulting in tumor hypoxia and lack of nutrients, and finally inhibit the appreciation of tumor cells [39, 40]. Although TACE can reduce the volume of primary liver cancer and improve the condition of patients in a short time, it will lead to increased metastasis of lymph nodes, lungs, and bones. Moreover, TACE may not be the best treatment for patients with extensive tumor bulk, multi-nodular spread, or impaired liver function. Thus, identifying new key factors that regulating angiogenesis will offer better therapeutic effect to the outcome of HCC. Herein, our study demonstrates that MYBL1 is overexpressed and correlates with tumor microvascular density and with distant metastasis in HCC, suggesting that upregulation of MYBL1 might contribute to the malignant potential of HCC. We found that overexpression of MYBL1 dramatically enhanced, whereas downregulation of MYBL1 reduced angiogenesis in HCC. Therefore, our results suggest that MYBL1 functions as a critical pro-angiogenic factor in HCC by performing both in vitro and in vivo approaches, and that MYBL1 may represent a novel prognostic biomarker and anti-angiogenesis target for HCC patients.

Amongst all cancer subtypes, gastrointestinal malignancy (esophageal, gastric, colorectal, pancreatic, and liver) are responsible for the most common and lethal solid tumors in the worldwide [41, 42]. Nowadays, the treatment of gastrointestinal cancer is still the conventional therapeutic approaches such as surgery, chemotherapy, and radiation therapy, which are indispensable but insufficient. Despite the advances in immunotherapy and other conventional treatment strategies, the 5-year survival prognosis and recurrence-free survival prognosis of tumor patients are still unsatisfactory [43]. In most of the cases, the limitation of the prognosis of patients with malignant gastrointestinal tumors stems from late diagnosis, underscoring the need for early detection and intervention. Therefore, identifying effective diagnostic markers and delineation of the mechanisms may provide new clues for the development of targeted cancer therapies, subsequently may lead to an increase in overall survival rates of patients. Interestingly, in our study we found that MYBL1 was not only overexpression in HCC but also in other gastrointestinal malignancy (pancreatic cancer, esophageal carcinoma, stomach adenocarcinoma, colon cancer), and upregulation of MYBL1 induces angiogenesis promotes HCC cells metastasis. More importantly, we found that the clinical relevance of MYBL1 and ANGPT2 was further confirmed in multiple gastrointestinal tumors. These results further support the notion that MYBL1 might be a valuable early predictor for prognosis in patients with gastrointestinal malignancy. It would be worthy to further investigate whether MYBL1 plays a role in promoting angiogenesis and metastasis in others gastrointestinal tumors.

## Supplementary information

SUPPLEMENTAL MATERIAL

Supplementary Information

checklist

## Data Availability

The datasets used and/or analyzed during the current study are available from the corresponding author upon reasonable request.
