# Supplementary figures and images for: MYBL1 induces transcriptional activation of ANGPT2 to promote tumor angiogenesis and confer sorafenib resistance in human hepatocellular carcinoma
Source: Cell Death Dis. 2022 Aug 20;13(8):727. doi: 10.1038/s41419-022-05180-2 (PMC9392790; doi:10.1038/s41419-022-05180-2)

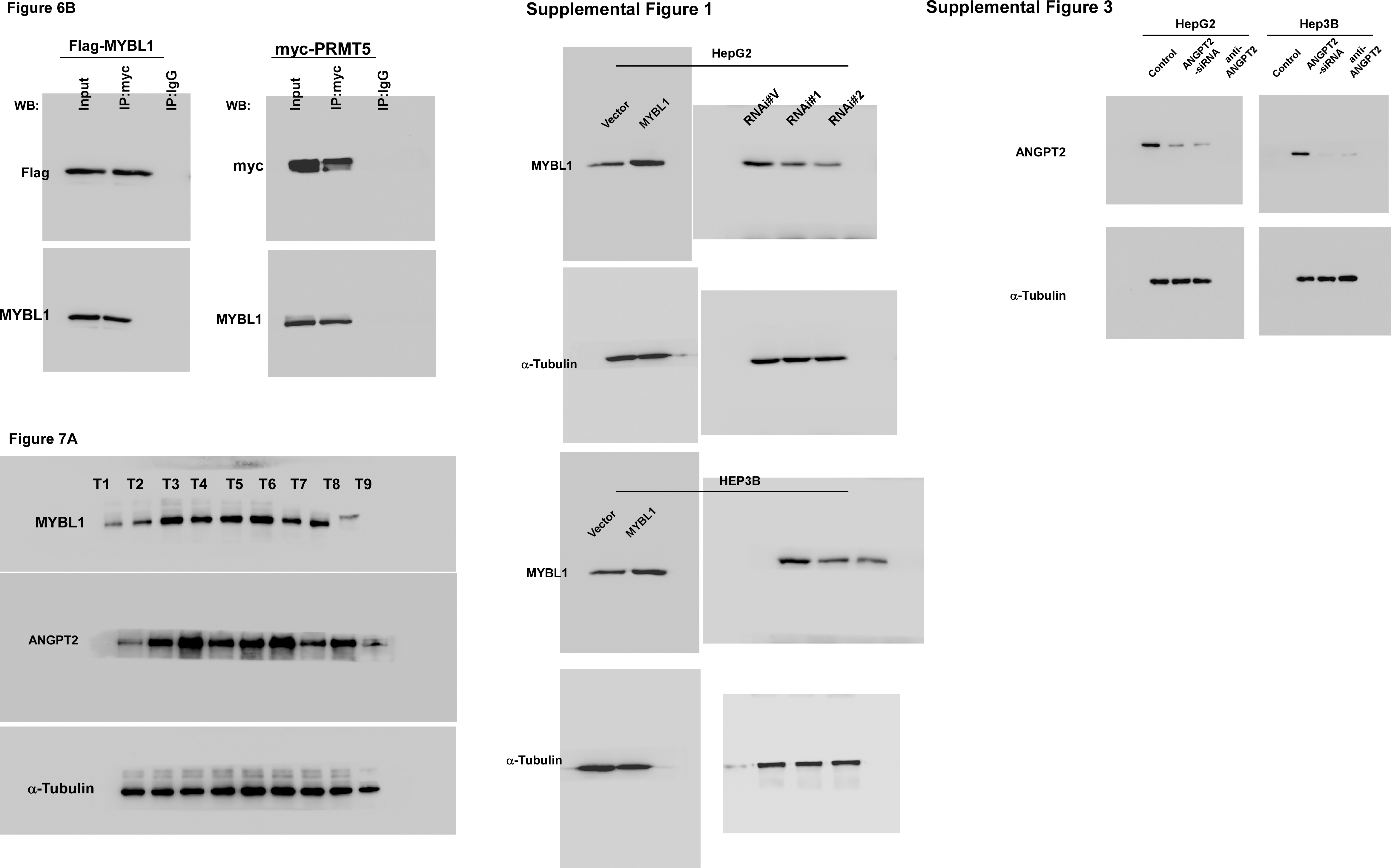

Supplement: Supplementary file 1 — SUPPLEMENTAL MATERIAL [file 41419_2022_5180_MOESM1_ESM.tif]
