# Supplementary material for: MYBL1 induces transcriptional activation of ANGPT2 to promote tumor angiogenesis and confer sorafenib resistance in human hepatocellular carcinoma
Source: Cell Death Dis. 2022 Aug 20;13(8):727. doi: 10.1038/s41419-022-05180-2 (PMC9392790; doi:10.1038/s41419-022-05180-2)
Supplement: Supplementary file 2 — Supplementary Information [file 41419_2022_5180_MOESM2_ESM.doc]

**Supplemental Information**

**Materials and Methods**

**Animal studies**

The BALB/c-nu mice used in this study were purchased from the Center of Experimental Animal of Guangzhou University of Chinese Medicine. The mice were 5 to 6 weeks old and randomly divided into 4 groups (*n =* 5/group). Each group of mice was inoculated subcutaneously with HepG2/Vector cells (1×10^6^), HepG2/MYBL1 cells (1×10^6^), HepG2/Control cells (1×10^6^), HepG2/MYBL1-RNAi cells (1×10^6^), in the right dorsal flank per mouse. Tumors were examined twice weekly; length and width measurements were obtained with calipers and tumor volumes were calculated using the equation larger diameter x (smaller diameter)2/2. Tumors were detected by an IVIS imaging system once every 5 days, and animals were euthanized, tumors were excised, weighed and paraffin-embedded. In the orthotropic tumor model, the indicated cells (1 × 10^6^)) were injected into the liver of mice. Sorafenib (1.25 mg/kg/day) were injected intraperitoneally every 5 days for 4 weeks. On day 28, animals were euthanized, tumors or liver were excised, weighed and paraffin-embedded. Serial 5.0 μm sections were cut and subjected to IHC staining using an anti-CD31, anti-Ki67, anti-Tunel and anti-MYBL1 antibodies. The animal experimental procedures of our study have been approved by the Institutional Animal Care and Use Committee of Guangdong Pharmaceutical University.

**Migration assay**

HCC cells in logarithmic growth stage were digested with 0.25% trypsin and resuspended with serum-free DMEM medium. 1 × 10^4^ cells were cultured inoculation in Transwell filter. The medium in the chamber was 200 μ l serum-free DMEM. Add 500ul DMEM medium containing 10% fetal bovine serum into the outside room and continued to culture for 24 hours; then fixed the cells in the fixed solution (methanol: glacial acetic acid = 3:1) for 10minand and soak in hematoxylin for 5min. Count the cells of 10 visual fields under 200 times optical microscope and take the average value; Observe and take photos under the microscope. The number of cells passing through 10 visual fields was calculated for statistics.

**Invasion assay**

HCC cells in logarithmic growth stage were digested with 0.25% trypsin and resuspended with serum-free DMEM medium. 1 × 10^4^ cells were cultured inoculation in Transwell filter (pre-coated with Matrigel) (BD Biosciences). The medium in the chamber was 200 μ l serum-free DMEM. Add 500ul DMEM medium containing 10% fetal bovine serum into the outside room and continued to culture for 24 hours; then fixed the cells in the fixed solution (methanol: glacial acetic acid = 3:1) for 10minand and soak in hematoxylin for 5min. Count the cells of 10 visual fields under 200 times optical microscope and take the average value; Observe and take photos under the microscope. The number of cells passing through 10 visual fields was calculated for statistics.

**Luciferase assay**

Three thousand cells were seeded in triplicate in 48-well plates and allowed to settle for 24 h. Clean the cultured cells with 1x PBS and be careful not to make the cells fall off. Remove the cleaning fluid and add 1x lysis reagent (the Dual Luciferase Reporter Assay Kit, Promega) sufficient to cover the cells. Scrape off the adherent cells in the Petri dish, and then transfer the cells and all liquids to a centrifuge tube. Transient centrifugation (room temperature 12000) ×g centrifugation for 15 seconds or 4 ℃ for up to 2 minutes to precipitate the fragments, and then transfer the supernatant to a new tube. Mix 20 µ L cell lysate with 100 µ l luciferase detection reagent (LAR) to detect the luminous signal according to a protocol provided by the manufacturer.

**Tube formation assay.** Prepare the dissolved Matrigel and precool the 24-well plate at - 20 ℃. The 24-well plate is laid with 200ul Matrigel for standby. HUVEC (2×10^4^) cells were washed twice with 1 x SA and trypsin inhibitor was terminated. HUVEC cells were added to each well and incubated at 37°C in 5% CO_2_ for 20 h. Pictures were taken under a 100× bright-field microscope and the capillary tubes were quantified by counting length.

**Chicken chorioallantoic membrane (CAM) assay.** 5-day-old chicken embryos were purchased from Yueqin breeding Co. Ltd, Guangdong, China. Chicken embryos were carefully scrubbed with 1/1000 benzalkonium bromide and incubated in an incubator at 37 ° C for 4 days. During the incubation period, the cells were laid, and 2x10^5^ cells were laid in the six well plate. After 24 hours, they were replaced with phenol red and serum-free DMEM. After 24 hours, the supernatant of the target cells was collected and filtered with a filter. Take out the chicken embryo from the incubator at 37 ° C, wipe it with 75% alcohol cotton ball, and gently tap a small hole with scissors at the head of the air chamber; Add 100ul of freshly collected cell supernatant and place it in 37 incubators for 5 days. Take out the chicken embryo filter membrane, disinfect the inoculation site with 75% ethanol, peel off the transparent tape and its surrounding allantoic membrane, and wash the surface blood with 1x PBS. Pick it up with a glass slide and take photos. The small new blood vessels grow to form medium and coarse blood vessels and continue to branch out small blood vessels in the medium and coarse blood vessels to form a new blood vessel network.

***Enzyme-linked immunosorbent assay (ELISA)***

The concentration of ANGPT2 was determined using a commercially available Human Angiopoietin 2 ELISA Kit (Proteintech, Cat No: KE00226). ELISA was performed according to the manufacturer’s instructions.

**Chemical reagents.** WDR5 inhibitor (OICR-9429) PRMT5 inhibitor (EPZ015866) were purchased from MedChemexpress (Monmouth Junction, NJ, USA). Anti-ANGPT2 antibody was purchased from R&D (#623-AN-025)

**Chromatin Immunoprecipitation (ChIP) assay.**

The ChIP assay was performed by using immunoprecipitation (ChIPs) assay kit (Upstate/Millipore, Billerica, MA) according to the manufacturer's instructions. The chromatin supernatants were incubated with anti-MYBL1 (Affinity), or anti-PRMT5 (ab109451), anti-H3 (ab1791), anti-H3R2me1(ab15584), anti-H3R2me2s (ab194684), anti-H3R8me2s (ab130740), anti-H2AR3me2s (ab22397), anti-H4R3me2s (ab5823), anti-H3K4me3 (ab12209), anti-Flag and anti-HA (Sigma-Aldrich, St. Louis, MO, USA) antibodies or an anti-Anti-IgG antibody (a negative control, Millipore, Billerica, MA) respectively, overnight at 4°C. Free DNA was extraction by using the QIAquick PCR Purification Kit (QIAGEN) and PCR was performed using specific primers. PCR primers is indicated in supplemental Tables 3.

**Biotinylated deactivated Cas9 capture analysis**

Biotinylated deactivated Cas9 capture analysis was performed following the published protocol [1]. Briefly, HepG2 cells (5 × 10^7^)) which transfected with FB-dCas9 plasmid and ANGPT2 promoter sgRNAs were harvested when treated with 1% formaldehyde to cross-link the proteins to DNA. Anti-streptavidin antibody was used for incubated the cell lysates. The ChIP proteins were separated by SDS-PAGE gel, subsequencely analyzed by IP-MS analysis (Shenzhen weinafe Biotechnology Co., Ltd).

**Immunoprecipitation and mass spectrometry (MS) analysis.**

Immunoprecipitation and mass spectrometry analysis were performed according to our previous studies. Lysates from the indicated cells were incubated with HA-, or myc-, or Flag-conjugated agarose beads (Sigma-Aldrich, Germany). For the mass spectrometry (MS) analysis, specific bands were excised and subjected to LC-MS/MS analysis.

**References**

1． Liu X., Zhang Y., Chen Y. In situ capture of chromatin interactions by biotinylated dCas9. Cell. 2017;170:1028–1043.

**Supplemental Figure Legends**

**Supplementary Figure** 1. (A). Western blot analysis of MYBL1 in the indicated HCC cells. α-Tubulin was used as a loading control. (B). Quantification of indicated migration HCC cells by transwell assays. **(C).** Quantification of indicated invation HCC cells by transwell assays. **
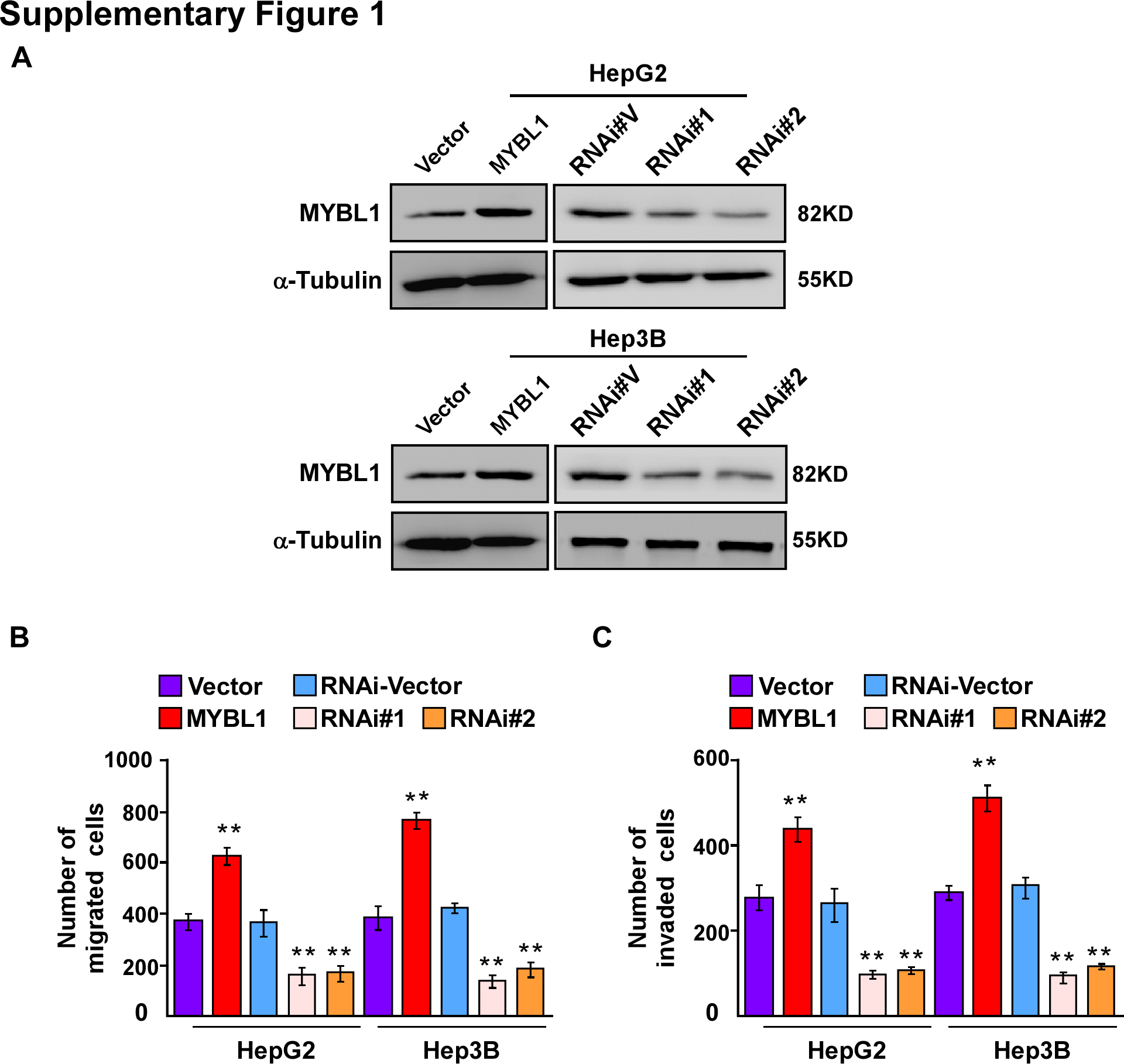
**

**Supplementary Figure** 2. (A-B) Relative mRNA expression (A) and protein level (B) of ANGPT2 in the indicated HCC cells, analyzed via Real-time PCR analysis and ELISA assay. Transcript levels were normalized to GAPDH expression. (C). Transactivation of the *ANGPT2* promoter in the indicated HCC cells, as demonstrated by luciferase activity assay. (D). Transactivation activity of the indicated serial *ANGPT2* promoter fragments in the indicated Hep3B cells. Each bar represents the mean ± SD of three independent experiments. * **P* < 0.01.


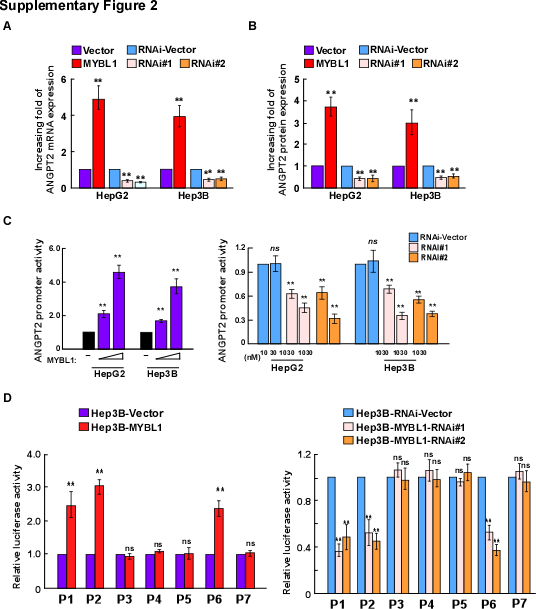


**Supplementary Figure** 3. Western blot analysis of ANGPT2 in the indicated HCC cells. α-Tubulin was used as a loading control.


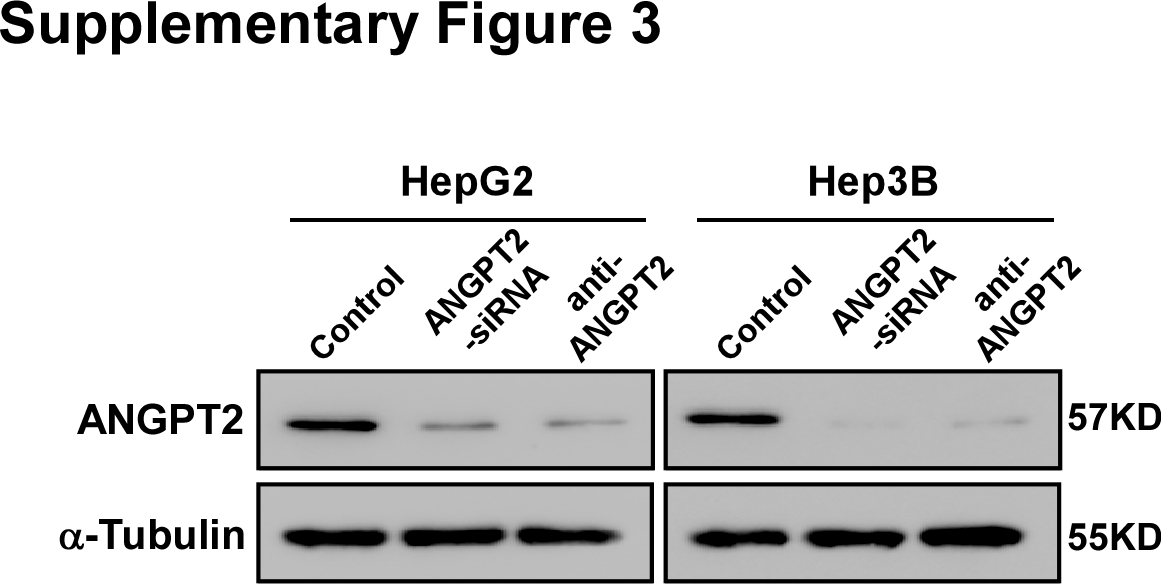


**Supplementary Figure** 4. (A) Relative mRNA expression of ANGPT2 in the indicated HCC cells, analyzed via Real-time PCR analysis. Transcript levels were normalized to GAPDH expression. (B). Quantification of indicated migration HCC cells by transwell assays. **(C).** Quantification of indicated invation HCC cells by transwell assays. **(D)** Quantification of HUVEC tube formation in the indicated cells (left); Cell migration assay was performed in the indicated cells (right). (E). Quantification of indicated migration HCC cells by transwell assays (left); Quantification of indicated invation HCC cells by transwell assays (right). Each bar represents the mean ± SD of three independent experiments. * *P* < 0.05.


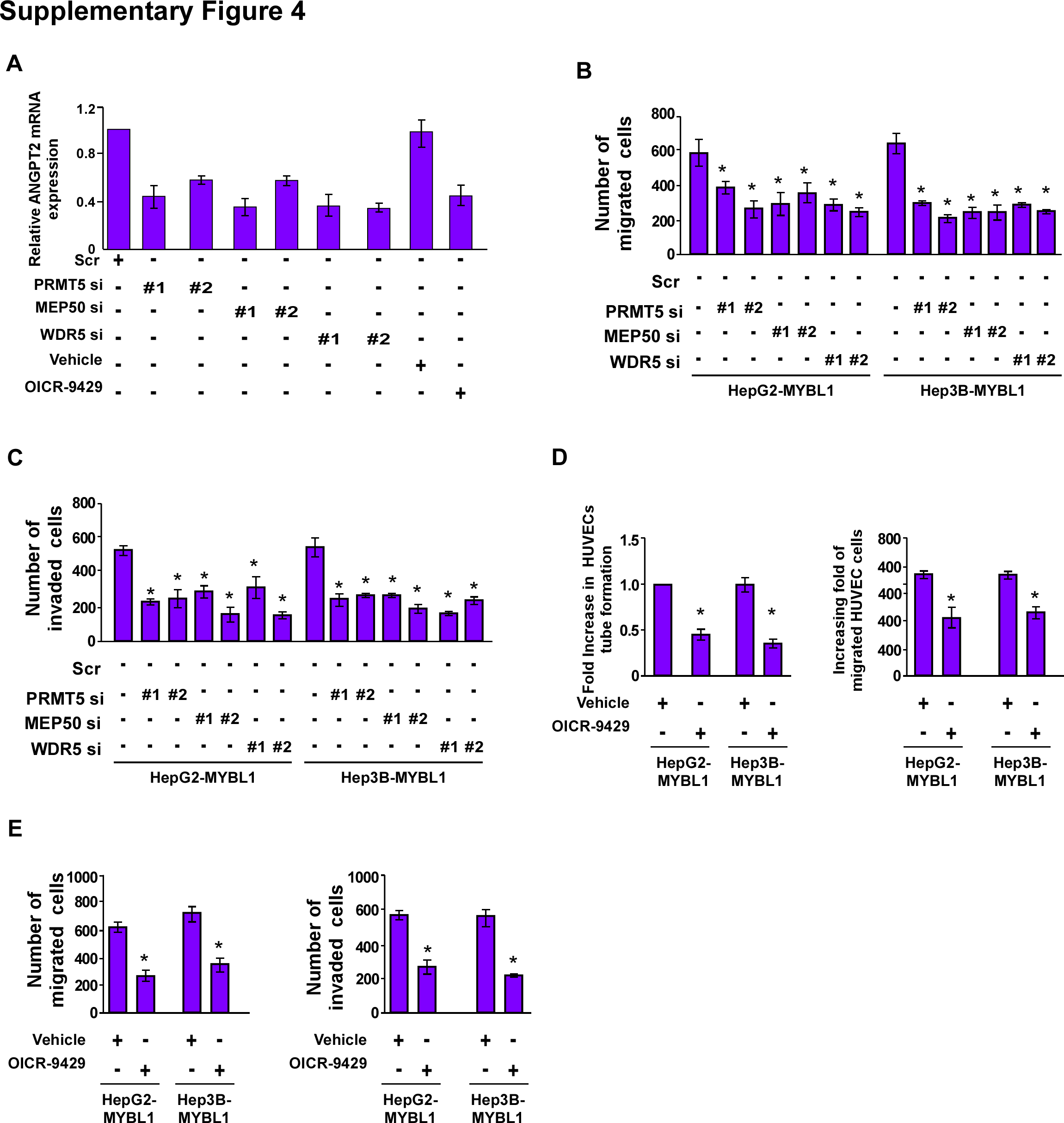


**Supplementary Figure** 5. Upregulated MYBL1 in several gastrointestinal cancers tissues (Tumor) compared with normal tissue (Normal). Pancreatic Cancer (NCBI/GEO/GSE 43795; n=31); Stomach adenocarcinoma (TCGA; n=446); Esophageal carcinoma (TCGA; n=199); Colon adenocarcinoma (TCGA; n=500).

**
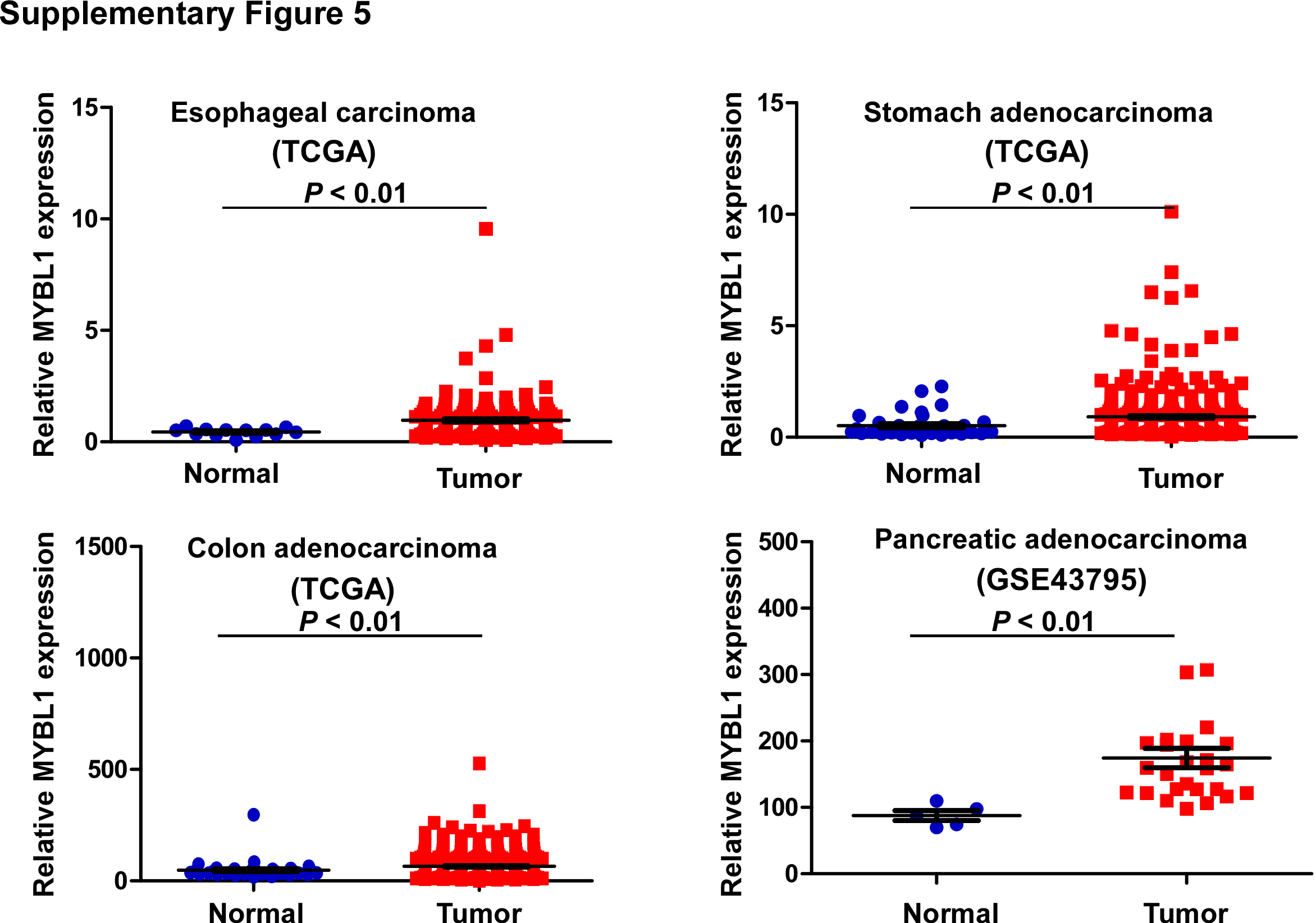
**

**Supplementary Table 1. The relationship of MYBL1 expression in 88 HCC with clinical pathological characteristics.**

|  |  |  | MYBL1 expression | |
| --- | --- | --- | --- | --- |
| Characteristics | Subgroup | Patients, Number(%) | Low(40) | High(48) |
| Gender |  |  |  |  |
|  | Male | 44(50) | 23 | 21 |
|  | Female | 44(50) | 17 | 27 |
| Age (years) |  |  |  |  |
|  | ≤50 | 53(60.2) | 33 | 20 |
|  | >50 | 35(39.8) | 7 | 28 |
| AFP, ng/mL |  |  |  |  |
|  | ≤20 | 50(56.8) | 25 | 25 |
|  | >20 | 38(43.2) | 15 | 23 |
| Liver cirrhosis |  |  |  |  |
|  | Absent | 14(15.9) | 8 | 6 |
|  | Present | 74(84.1) | 32 | 42 |
| Distant metastasis |  |  |  |  |
|  | NO | 28 (31.8) | 23 | 5 |
|  | YES | 60(68.2) | 17 | 43 |

**Supplementary Table 2. Univariate and multivariate analysis of different prognostic parameters in patients with HCC by Cox-regression analysis**

|  | **Univariate analysis** | | **Multivariate analysis** | |
| --- | --- | --- | --- | --- |
|  | ***P*** | **Hazard ratio**  **(95% CI)** | ***P*** | **Hazard ratio**  **(95% CI)** |
| **Gender** | 0.647 | 0.820  (0.350-1.921) | 0.947 | 1.031  (0.415-2.565) |
| Male |  |  |  |  |
| Female |  |  |  |  |
| **Age (years)** | 0.105 | 2.066  (1.179-3.621) | 0.247 | 1.621  (0.716-3.668) |
| ≤50 |  |  |  |  |
| >50 |  |  |  |  |
| **AFP, ng/mL** | 0.384 | 1.250  (0.756-2.0467) | 0.157 | 1.510  (0.853-2.673) |
| ≤20 |  |  |  |  |
| >20 |  |  |  |  |
| **Liver cirrhosis** | 0.034 | 1.358  (1.040-1.801) | 0.742 | 0.932  (0.612-1.42) |
| Absent |  |  |  |  |
| Present |  |  |  |  |
| Distant metastasis | 0.014 | 1.728  (1.143-3.275) | 0.001 | 2.129  (1.541-6.128) |
| Yes |  |  |  |  |
| NO |  |  |  |  |
| **MYBL1 level** | 0.001 | 4.225  (1.806-9.886) | < 0.001 | 15.397  (4.296-55.182) |
| Low expression |  |  |  |  |
| High expression |  |  |  |  |

**Supplementary Tables 3 Primers and Oligonucleotides used in this study.**

| **Primer used for subcloning and plasmid construction:** | |
| --- | --- |
| **MYBL1-forwards** | gccATGGATTACAAGGACGACGATGACAAGATGGCGAAGAGGTCGCGCAG |
| **MYBL1-downwards** | gccTTACAGTATGAGAGCTCTTG |
| **Primer used for real-time RT-PCR** | |
| MYBL1-up | AGGCAAGCAGTGTAGAGAAAGA |
| MYBL1-dn | CGATTTCCCAACCGCTTATGT |
| GAPDH-up | GGAGCGAGATCCCTCCAAAAT |
| GAPDH-dn | GGCTGTTGTCATACTTCTCATGG |
| ANGPT2-up | AGGCTGCAAGTGCTGGAGAA |
| ANGPT2-dn | GCTGTTTGGTTCAACAGGT |
| **Oligonucleotides for siRNAs** | |
| MYBL1 siRNA#1 | TTTGGCAATTTCTGCCCAACG |
| MYBL1 siRNA#2 | TAATGAGAGCTTTCTGCCCAC |
| ANGPT2 siRNA | GAUCGAGAUUGGAACCAGUTT |
| PRMT5 siRNA | CCCATCCTCTTCCCTATTAAG |
| MEP50 siRNA | GAGAGGTATTCTAGTGGCCTCCGAT |
| WDR5 siRNA | TGGTCGTCAGATTCTAACCTT |
| **ANGPT2 promoter primers used for ChIP assay** | |
| F1-forward | 5’ TAGTATGATTCCACCAAGG-3’ |
| F1-reverse | 5’ CATAAGATGGAGATCAT -3’ |
| F2-forward | 5’ TGTCTTCTTTTGAGAAGG-3’ |
| F2-reverse | 5’ AGGTTCTGGACATGGTTT -3’ |
| F3-forward | 5’ TTTTTACTAAGCTCTAACCG -3’ |
| F3-reverse | 5’ AGAAGGTGTTTATAGGC-3’ |
| F4-forward | 5’ TTAACTCAGCAGCCCAAGT -3’ |
| F4-reverse | 5’ ATTTTTTACTAAGCTCTAAC-3’ |
| F5-forward | 5’ ATGTCCTCATCTGTGCAATTC-3’ |
| F5-reverse | 5’ AGCCCAAGTGTACATAGGGA-3’ |
| F6-forward | 5’ CTTCTTTATTGTGTCAGA-3’ |
| F6-reverse | 5’ CCTCATCTGTGCAATTCAAG -3’ |
| F7-forward | 5’ TTCTCTTGCCTCAGGCTCCTG-3’ |
| F7-reverse | 5’ CTTCTTTATTGTGTCAGAATTTGTTG-3’ |
| F8-forward | 5’ AAAAACTCCACTTGGATATTC-3’ |
| F8-reverse | 5’ CTCTTGCCTCAGGCTCCTGAG -3’ |
| F9-forward | 5’ TATTACAAATAGGTCAGGA-3’ |
| F9-reverse | 5’ AAAAAACTCCACTTGGATATTCAC -3’ |
| F10-forward | 5’ AAAAACCGATCTGTTACAGGA-3’ |
| F10-reverse | 5’ AAAATATTACAAATAGGTCAGGACT-3’ |
| F11-forward | 5’ TCCTTTCCTTATATGATAAGT-3’ |
| F11-reverse | 5’ AAAACCGATCTGTTACAGGAC-3’ |
